# The Incidence of Cancer Is Increased in Hospitalized Adult Patients With Obstructive Sleep Apnea in China: A Retrospective Cohort Study

**DOI:** 10.3389/fonc.2022.856121

**Published:** 2022-03-31

**Authors:** Hailin Xiong, Miaochan Lao, Longlong Wang, Yanxia Xu, Guo Pei, Bin Lu, Qianping Shi, Jialian Chen, Shuyi Zhang, Qiong Ou

**Affiliations:** ^1^ The Second School of Clinical Medicine, Southern Medical University, Guangzhou, China; ^2^ Sleep Center, Department of Pulmonary and Critical Care Medicine, Guangdong Provincial People’s Hospital, Guangdong Academy of Medical Sciences, Guangdong Provincial Geriatrics Institute, Guangzhou, China; ^3^ Huizhou Municipal Central Hospital of Guangdong Province, Huizhou, China

**Keywords:** obstructive sleep apnea, cancer, incidence, mortality, lung cancer

## Abstract

**Background:**

The association between obstructive sleep apnea (OSA) and the incidence and mortality of cancer remain unclear, especially in Asian populations. Thus, this study was conducted to explore the relationship between OSA and the incidence and mortality of cancer in hospitalized patients.

**Methods:**

This retrospective cohort study evaluated inpatients from Guangdong Provincial People’s Hospital for suspected OSA between January 2005 and December 2015. Cancer incidence, all-cause mortality, and cancer mortality and were determined using data from the hospital information system and Centers for Disease Control. Between-group comparisons were carried out by performing a chi-square test and analysis of variance. Kaplan–Meier analysis and the Cox proportional risk model were applied to investigate the association between OSA and cancer incidence and mortality.

**Results:**

Of the 4,623 hospitalized patients included, 3,786 (81.9%) patients were diagnosed with OSA. After a median follow-up of 9.1 years (interquartile range, 9.79–11.44), the incidence of cancer was 6.6% (251/3,786), with lung cancer having the highest incidence at 1.6% (60/3,786). The mortality rate of OSA patients was higher than that of non-OSA patients (16.83% vs.12.78%, p=0.008), but the relationship between apnea-hypopnea index (AHI), oxygen saturation less than 90% (TSat90), and cancer mortality was not statistically significant (p>0.05).The mortality rate for all types of cancer was 2.8% (105/3,786), with lung cancer having the highest mortality rate at 0.8% (32/3,786). The cumulative incidence of cancer in the severe OSA group was 8.2%, which was higher than that in the normal, mild, and moderate OSA groups (*P*=0.010). Further, the Cox proportional risk regression model showed a progressive enhancement in the risk of cancer incidence as the AHI increased (adjusted hazard ratio [HR]: 1.009 [95% confidence interval (CI): 1.003–1.016], *P*=0.005). Based on subgroup analysis, the risk of cancer increased as the AHI increased in patients aged <65 years (adjusted HR: 1.019 [95% CI: 1.007–1.031], *P*=0.002). In addition, the cancer incidence was significantly higher in the severe OSA group than in the normal, mild, and moderate OSA groups (adjusted HR: 2.825 [95% CI: 1.358–5.878], *P*=0.019).

**Conclusion:**

The incidence of cancer is higher in patients with OSA than in non-OSA patients and is significantly positively associated with the severity of OSA. Particularly, for OSA patients aged <65 years, lung cancer is the main cause of death in those with new-onset cancer. Mortality was higher in OSA patients than in non-OSA patients.

## 1 Introduction

Obstructive sleep apnea (OSA) refers to a systemic disease categorized by intermittent hypoxia and sleep fragmentation caused by recurrent collapse of the upper airway during sleep, leading to apnea and/or hypoventilation (1). Approximately 1 billion people worldwide have OSA, with nearly 176 million (13.0%) of these OSA patients being in China (2). As an important risk factor, OSA is independently related to metabolic diseases, hypertension, and cardiovascular disease (3–6). Intermittent hypoxia and sleep fragmentation are hallmark features of OSA, and hypoxia-inducible factor-1 (HIF-1) exerts a vital function as a signaling factor in hypoxia-related events (7). The resulting oxidative stress, systemic inflammatory response, sympathetic hyperactivity, altered immune function, enhanced angiogenesis, and stromal cell support could be potential mechanisms for activation of oncogenic pathways (8–10). Intermittent hypoxic mouse models of OSA have shown enhanced cancer growth, invasion, and metastatic potential (11, 12). However, clinical studies on the correlation between OSA and oncological diseases are still scarce, and the relationship between them is poorly understood.

Two large retrospective cohort studies showed high cancer incidence in patients undergoing OSA and a positive correlation between cancer and OSA severity (13, 14). Further, although two clinical studies from the United States and Canada found no significant association between OSA, they found an enhanced risk of new-onset cancers (15, 16). According to a meta-analysis, OSA is associated with an increased overall incidence of cancer (17). In addition, another study found that OSA was independently associated with the incidence of all types of cancer, with a higher risk of cancer in moderate-to-severe OSA (18). Considering the differences in the design, methodology, and sample size of these studies as well as confounding factors, it can only be tentatively concluded that OSA may increase the risk of cancer incidence. Nevertheless, it is difficult to speculate an independent correlation. Studies addressing OSA and cancer incidence have varying degrees of limitations, such as the lack of information on some clinical parameters (14) and patient oxygen saturation indices (13), data on potential confounders of the relationship between OSA and cancer (16, 19, 20), and the lack of statistical power to analyze OSA and site-specific cancer incidence (21).

Other factors can also affect the results of studies on the correlation between OSA and the risk of tumor mortality. Tumor type, malignancy, treatment, and treatment modalities are often difficult to include in the analysis. It remains unclear whether the causes of death of patients in the Busselton community (22) and Wisconsin cohort (23) were related to tumors or OSA. A meta-analysis also concluded that there was significant heterogeneity between studies and that it is not yet possible to conclude that OSA is related to cancer morbidity and mortality (24). Moreover, most of the current studies are from sleep cohorts in Europe and the United States, and there are few studies on the Chinese population in mainland China, despite the high incidence of OSA in China. Thus, this study aimed to explore the relationship between OSA and cancer incidence and mortality in a Chinese cohort.

## 2 Materials and Methods

### 2.1 Study Design and Subjects

**Figure 1:**
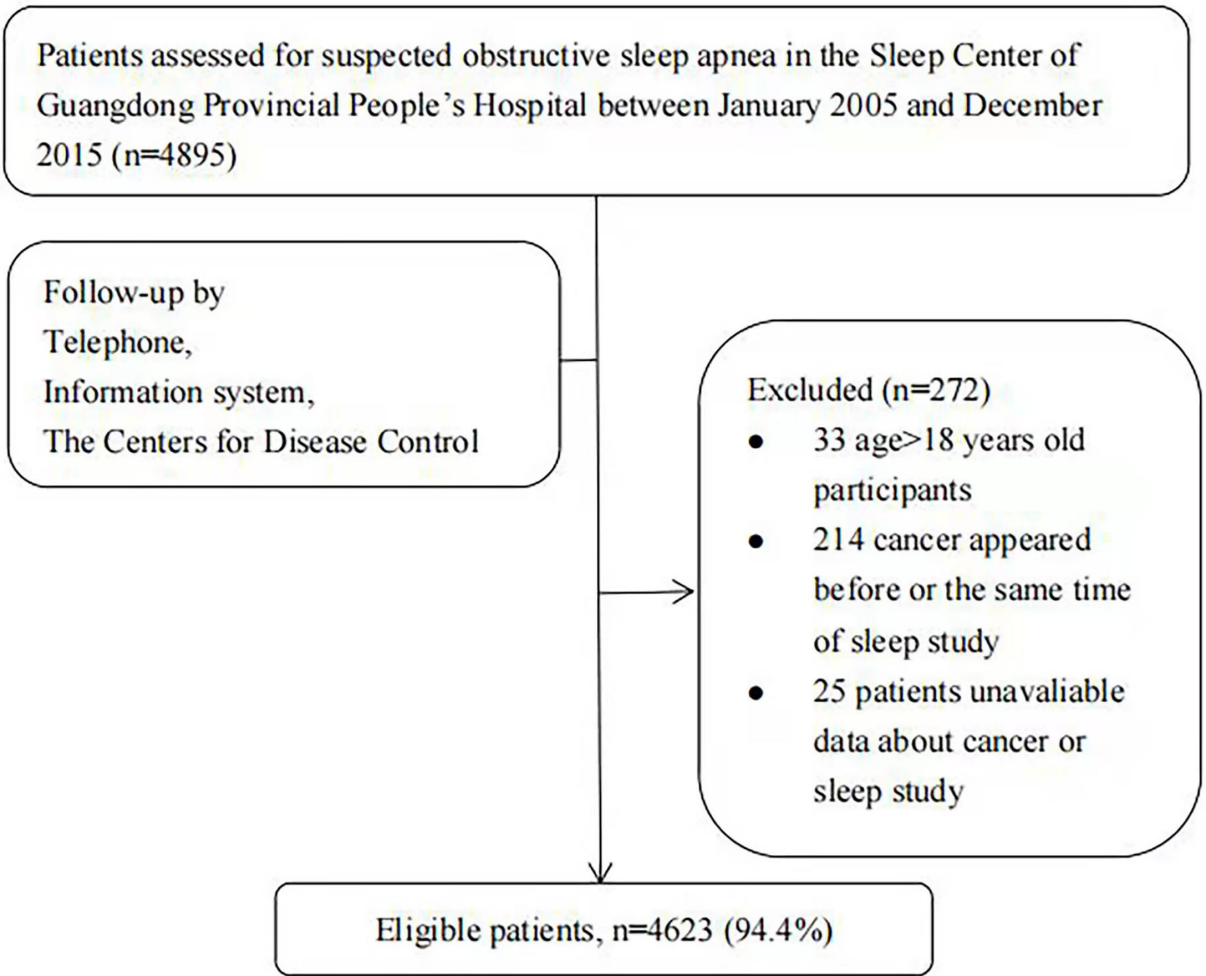
Patient inclusion flowchart.

This retrospective work evaluated 4,895 inpatients with suspected OSA who completed sleep monitoring between January 1, 2005, and December 31, 2015, at the Sleep Center of Guangdong Provincial People’s Hospital. The exclusion criteria were (1) cancer diagnosis before or within 6 months of sleep monitoring (15); (2) age <18 years; and (3) incomplete information on sleep monitoring or cancer. In total, 4,623 patients (94.4%) were contained in the analysis (**Figure 1**).

This study was approved by the Ethics Committee of Guangdong Provincial People’s Hospital (GDREC2019757H).

### 2.2 Data Collection

Baseline data were obtained from the medical information system and sleep center medical records, sleep monitoring reports, and scales at Guangdong Provincial People’s Hospital. Specifically, data on age (years), sex, body mass index (BMI, kg/m^2^), and smoking history (yes/no) were obtained from the sleep center patient medical record system. Meanwhile, common comorbidities at the time of sleep monitoring (e.g., hypertension, coronary heart disease, and diabetes) were determined from the hospital inpatient medical record system.

### 2.3 Sleep Monitoring

Daytime sleepiness was evaluated based on the Epworth Sleepiness Scale (ESS). Following the American Academy of Sleep Medicine Clinical Practice Guidelines for Diagnostic Testing of Obstructive Sleep Apnea in Adults (1), all patients with suspected OSA underwent primary sleep screening or polysomnography with sleep-related information containing nasal-oral airflow, chest wall and abdominal wall motion, arterial oxygen saturation, and apnea-hypopnea index (AHI) data as well as percent nighttime with oxygen saturation less than 90% (TSat90).

Sleep apnea was defined as the absence or significant reduction (90% decrease from baseline) of oronasal airflow during sleep for over 10 seconds. AHI referred to the number of apneas per hour of sleep and hypoventilation. OSA severity was categorized in accordance with the AHI value (25) as none (<5), mild (5–14.9), moderate (15–30), or severe (>30).

### 2.4 Study Endpoints

The primary endpoint was the incidence of all cancers, while the secondary endpoints were all-cause mortality and cancer-related mortality. Survival time was calculated from the date of study inclusion to death or the last follow-up. The patients were followed up for at least 6 months after the start of sleep monitoring, with the follow-up period ending on December 31, 2020. Cancer incidence was defined as the first occurrence of cancer at any time between >6 months after the start of the sleep study (15) and the final follow-up date. Cancer incidence and survival status were evaluated through the hospital information system and the National Cause of Death Registry Information Network system. The patients were followed up manually by telephone.

### 2.5 Statistical Analysis

AHI and TSat90, which are both continuous variables, were included as the main proxy variables for OSA (13). Normally distributed constant variables were denoted as the mean ± standard deviation, and between-group differences were explored based on the *t*-test or analysis of variance. Meanwhile, non-normally distributed continuous variables were expressed as medians and quartiles, and between-group differences were analyzed using nonparametric tests. The differences between groups for qualitative variables were tested by performing the chi-square test.

The Schoenfeld residual method was adopted for determining whether the variables satisfied the proportional hazards (PH) assumption, and the results confirmed that the variables met the PH assumption condition.

The correlation test results were consistent with the results of the Schoenfeld residual method. Kaplan–Meier survival curves and Cox proportional risk regression models were applied to evaluate the associations of cumulative cancer incidence, all-cause mortality, and cancer mortality with different OSA severities, with adjustment for potential confounders such as age, sex, BMI, and smoking status. In addition, subgroup analyses by sex (male vs. female) and age (<65 vs. ≥65 years) were also performed to assess differences in cancer incidence according to OSA severity. The test level for the current work was α=0.05, and *P ≤* 0.05 was regarded to show statistical significance. All statistical analyses were carried out based on SPSS software (version 23.0; SPSS Inc., Chicago, IL, USA).

## 3 Results

### 3.1 Patient Characteristics

The mean patient age 62.6 ± 14.5 years, 80.7% were male, 27.7% had a history of smoking, and the mean ESS score was 4.6 ± 4.0. In total, 3,786 patients were diagnosed with OSA; among them, 1,644 (36.0%), 1,064 (23.0%), and 1,058 (22.9%) had mild, moderate, and severe OSA, respectively. At the time of sleep monitoring, the top five most common comorbidities were hypertension (61.5%), coronary heart disease (52.4%), type 2 diabetes (25.9%), benign prostatic hyperplasia (BPH) (16.7%), and hyperlipidemia (10.1%). As shown in **Table 1**, the severe OSA group had higher mean BMI and included more male patients. There existed obvious differences among groups with respect to the rate of hypertension, coronary heart disease, type 2 diabetes, BPH, and hyperuricemia (**Table 1**).

**Table 1:** Baseline patient characteristics according to the severity of OSA.

Variables	Non-OSA (n=837)	Mild OSA (n=1664)	Moderate OSA (n=1064)	Severe OSA (n=1058)	*F*/*χ^2^*	*P*
Age, years	58.6±14.7	64.1±14.0	65.0±13.7	61.0±15.2	42.577	<0.001
Male sex	618 (73.8)	1320 (79.3)	876 (82.3)	919 (86.9)	55.048	<0.001
BMI, kg/m^2^	23.3±3.4	24.4±3.5	25.2±3.5	26.9±4.3	169.263	<0.001
Smoker	245 (29.3)	450 (27.0)	277 (26.0)	307 (29.0)	3.782	0.286
Hypertension	401 (47.9)	973 (58.5)	706 (66.4)	765 (72.3)	134.546	<0.001
CHD	447 (53.4)	930 (55.9)	588 (55.3)	459 (43.4)	46.443	<0.001
Type 2 diabetes	147 (17.6)	421 (25.3)	291 (27.4)	337 (31.9)	51.361	<0.001
BPH	101 (12.1)	285 (17.1)	195 (18.3)	189 (17.9)	16.217	<0.001
Hyperlipidemia	63 (7.5)	140 (8.4)	121 (11.4)	142 (13.4)	26.112	<0.001
TSat 90%	0.6±5.2	1.3±6.0	3.5±9.3	12.4±15.7	340.067	<0.001
TSat 90 mins	0.0 [0, 0]	1.0 [0, 0]	4.4 [0.8, 13.3]	30.4 [9.5, 77.1]	2001.086	<0.001
ESS score	2.7±2.2	4.3±5.1	3.1±2.0	4.9±3.6	68.90	<0.001

Data are presented as n (%), median [range], or the mean ± standard deviation (SD). AHI, apnea-hypopnea index; BMI, body mass index; ODI, oxygen desaturation index; TSat90, percent nighttime with oxygen saturation < 90%; ESS, Epworth sleepiness score; CHD, coronary heart disease; BPH, benign prostatic hyperplasia.

### 3.2 Follow-Up Outcomes

#### 3.2.1 Incidence and Types of New-Onset Cancer

**Figure 2:**
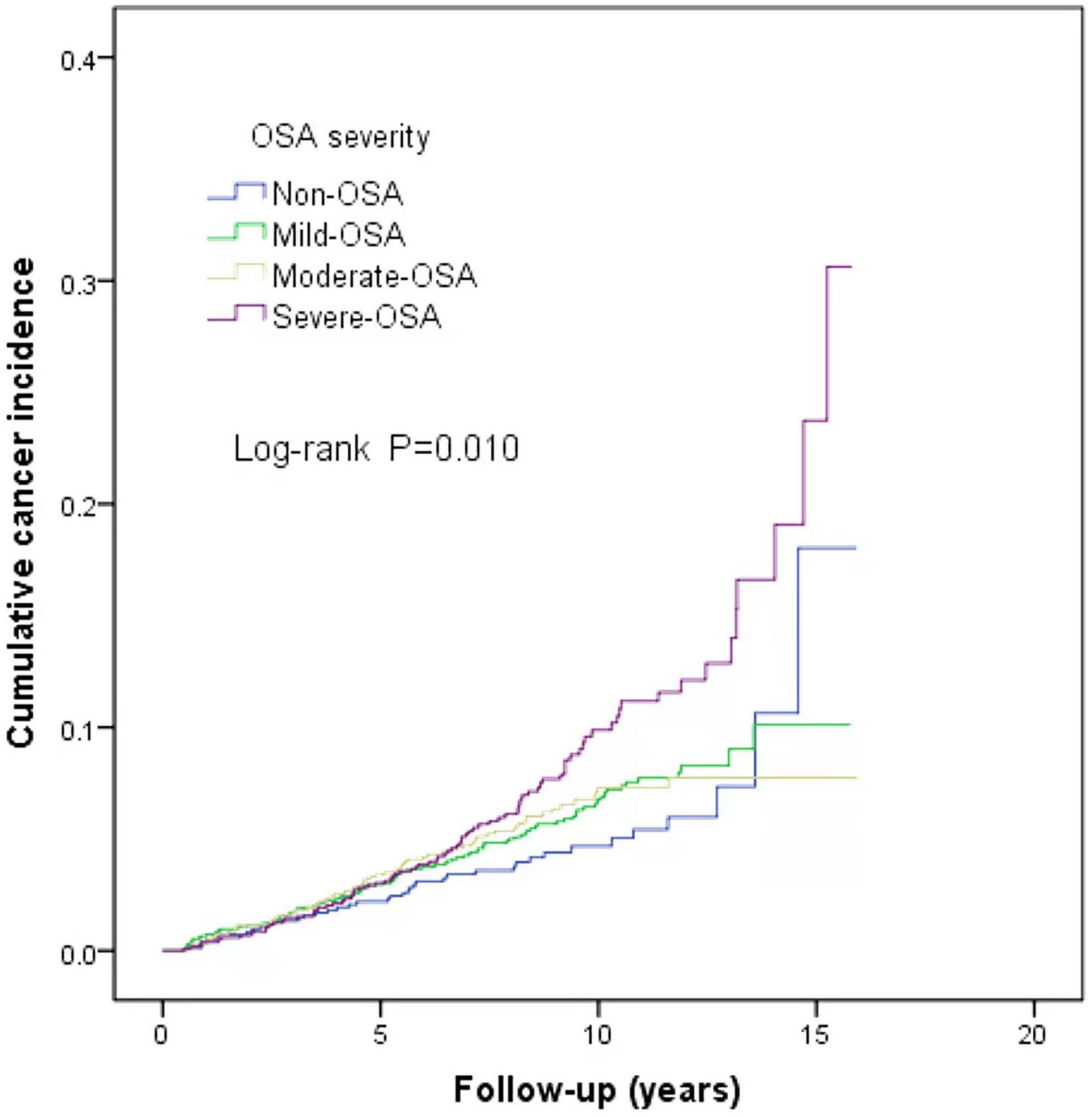
Kaplan–Meier curves of cumulative cancer incidence according to the severity of OSA.

**Figure 3:**
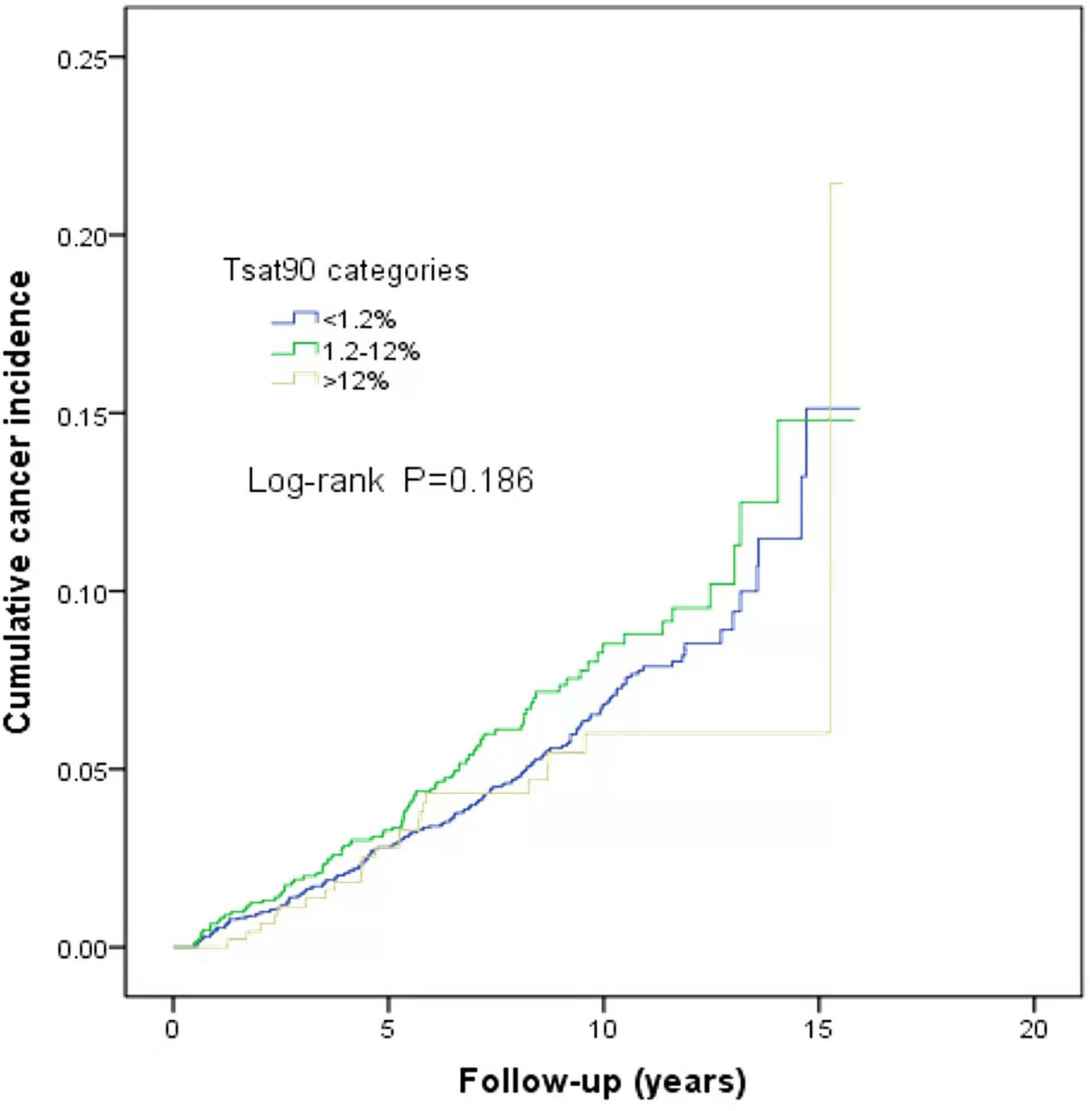
Kaplan–Meier curves of the cumulative cancer incidence according to the TSat90 categories.

This study is a long-span end-point follow-up.The median follow-up time was 9.1 years (interquartile range: 9.79–11.44 years), and by the end of follow-up, 290 patients (6.3%) were diagnosed with cancer. The median sleep monitoring time for patients diagnosed with cancer was 5.4 years (interquartile range: 3.00–8.15 years). The cancer incidence rate was 6.5% (243/3,733) in men and 5.3% (47/890) in women. The mean age of the cancer patients was 71.9 ± 11.5 years. The cancer incidence rates differed among the normal, mild, moderate, and severe OSA groups (χ^2^ = 10.890, P=0.012). The cumulative cancer incidence was higher in the severe OSA group than in the other groups: normal, 4.7% (39/837); mild, 6.1% (101/1,664); moderate, 5.9% (63/1,064); and severe, 8.2% (87/1,058).The cumulative incidence of all types of cancer was 6.6% (251/3,786), with lung cancer having the highest incidence at 0.8% (32/3,786) (**Figure 2**). There was no significant association between TSat90 and cancer incidence (log-rank *P*=0.186) (**Figure 3**). The top five most common types of new-onset cancers were lung, colorectal, prostate, stomach, and head and neck cancers (**Table 2**).

**Table 2:** Types of new-onset cancer.

Cancer type	New-onset cancers in the overall cohort, n (%)	New-onset cancers in OSA patients, n (%)	ICD-10 diagnostic codes
Lung cancer	70 (24.1)	60 (23.9)	C33, C34
Colorectal cancer	46 (15.9)	40 (15.9)	C18, C19, C20
Prostate cancer	45 (15.5)	42 (16.7)	C61
Gastric cancer	20 (6.9)	17 (6.8)	C16
Head and neck tumor	19 (6.6)	15 (6.0)	C01–C14
Malignant tumors of the liver and intrahepatic bile ducts	18 (6.2)	14 (7.2)	C22
Hematologic tumor	9 (3.1)	8 (3.2)	C82, C90, C91, C92
Breast cancer	8 (2.8)	7 (2.8)	C50
Urothelial carcinoma	8 (2.8)	6 (2.4)	C65, C66, C67
Pancreatic cancer	8 (2.8)	7 (2.8)	C2
Other cancers	39 (13.4)	35 (14.0)	
**All cancers**	290 (100)	251 (100)	

#### 3.2.2 All-Cause and Cancer Mortalities

**Figure 4:**
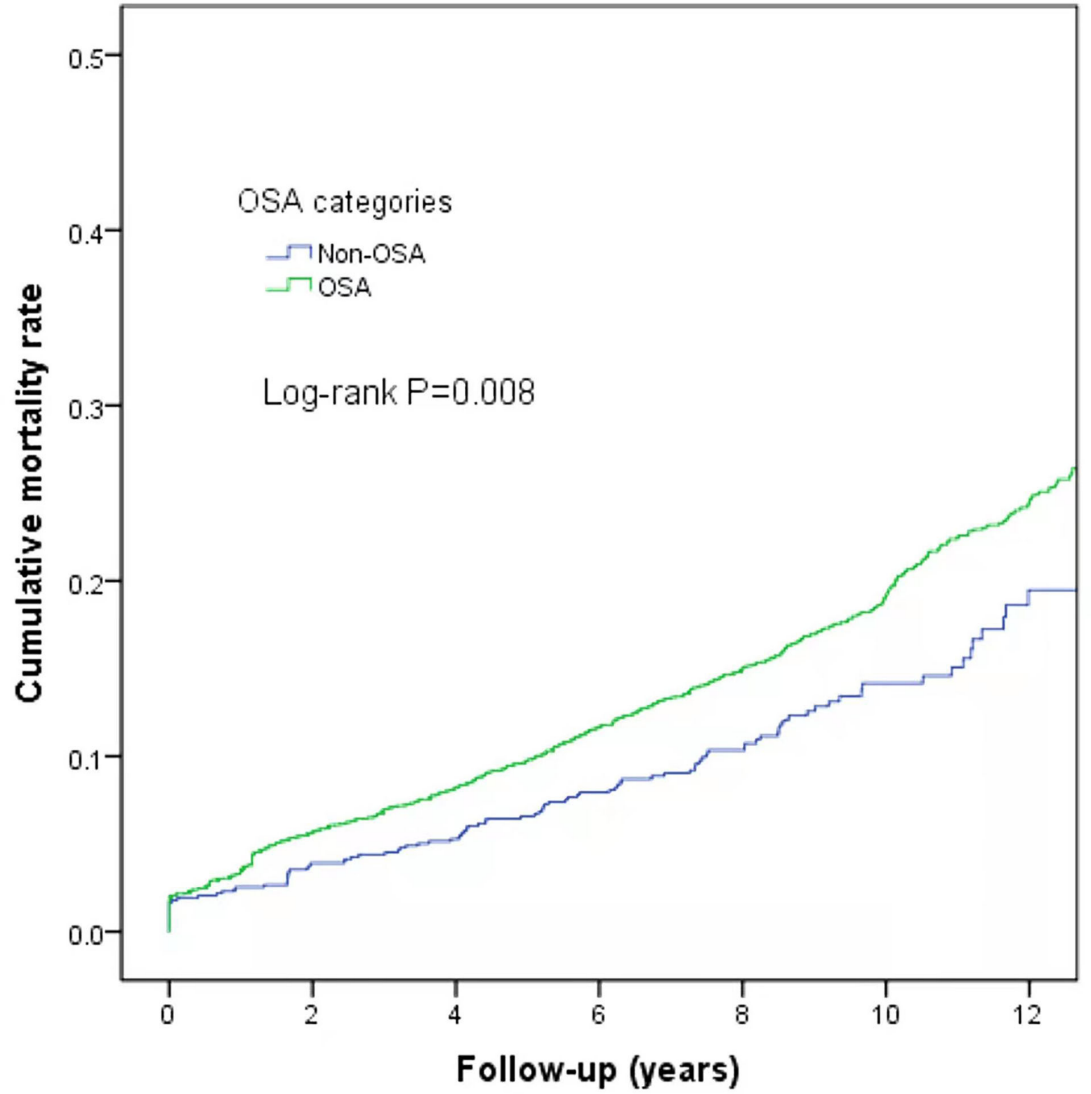
Kaplan–Meier curves of survival according to the severity of OSA.

At the end of follow-up, there were 744 deaths (16.09%) with differences in all-cause mortality among the normal, mild, moderate, and severe OSA groups (χ^2^ = 9.775, *P*=0.021); the all-cause mortality rates were 12.78% (107/837), 17.4% (290/1,664), 17.0% (181/1,064), and 15.7% (166/1,058), respectively. Further analysis of OSA as a dichotomous variable revealed a higher mortality rate in the OSA patients than in the non-OSA patients (log-rank P=0.008) (**Figure 4**).

In total, 123 patients died from cancer (16.5%), with lung cancer (n=39) being the most common cause of cancer-related death, followed by colorectal cancer (n=21) and hepatobiliary system tumors (n=14). There were 1-12 deaths from other types of cancer. The cancer-specific mortality rates for the patients with new-onset cancer in the normal, mild, moderate, and severe OSA groups were 46.2% (18/39), 44.6% (45/101), 44.4% (28/63), and 36.8% (32/87), respectively. There existed no obvious differences among the groups in terms of mortality rate among the cancer patients (χ^2^ = 1.649, *P*=0.648).

### 3.3 Association of OSA-Related Parameters With the Incidence and Mortality of Cancer

When AHI was contained as a continuous variable in the multivariate Cox regression analysis, the risk of cancer incidence increased as the AHI increased (adjusted hazard ratio [HR], 1.009; 95% confidence interval [CI]: 1.003–1.016, *P*=0.005). In addition, cancer incidence was higher in the severe OSA group when AHI was contained as a ranked variable in the multivariate Cox regression analysis based on an adjusted HR of 1.519 (95% CI: 1.039–2.222, *P*=0.031). Further, TSat90, either as a categorical or continuous variable, was not obviously related to cancer incidence (*P*>0.05) (**Table 3**). There existed no obvious association between AHI and all-cause or cancer mortality (*P*>0.05). All-cause mortality was higher in the TSat90 >12% group than in the TSat90 <1.2% group (adjusted HR: 1.296, 95% CI: 1.027–1.636, P=0.029), while there existed no significant correlation between TSat90 and cancer mortality (**Table 4**).

**Table 3:** Multivariate Cox regression analysis of the relationship between cancer incidence and AHI and TSat90%.

Category	Entire cohort (n=4623)	
	Adjusted HR (95% CI)	*P*
**AHI, events/h**		
AHI (continuous)	1.009 (1.003-1.016)	**0.005**
AHI categories		
Non-OSA	1	–
Mild- OSA	0.984 (0.679-1.427)	0.984
Moderate-OSA	1.024 (0.686-1.530)	0.906
Severe-OSA	1.519 (1.039-2.222)	**0.031**
**TSat90, %**		
TSat90 (continuous)	0.992 (0.980-1.004)	0.992
TSat90 categories		
<1.2%	1	
1.2-12%	1.111 (0.856-1.441)	0.430
>12%	0.756 (0.485-1.177)	0.215

Adjusted for sex, age, BMI, smoking status, hypertension, CHD, type 2 diabetes, BPH, and hyperuricemia.

AHI, apnea-hypopnea index; OSA, obstructive sleep apnea; TSat90, percentage of nighttime with oxygen saturation <90%. In bold: P = 0.005 indicated that when the AHI was included as a continuous variable in cox regression, the higher the AHI was, the higher the risk of cancer (HR=1 .009, 95%CI: 1.003-1.016). P = 0.031 indicated that when the AHI was included as a graded variable in cox regression analysis, the risk of cancer incidence in severe OSA patients was 1.519 times higher than that in non-OSA patient s (HR=1.519, 95%CI=1.039, 2.222).

**Table 4:** Multivariate Cox regression analysis of the association between mortality and OSA severity.

Categories	All-cause mortality		Cancer mortality	
	Adjusted HR (95% CI)	*P*	Adjusted HR (95% CI)	*P*
AHI categories				
Non-OSA	1	–	1	
Mild OSA	0.922 (0.738-1.151)	0.472	0.840 (0.485-1.454)	0.532
Moderate OSA	0.867 (0.681-1.102)	0.244	0.840 (0.463-1.527)	0.568
Severe OSA	0.965 (0.755-1.232)	0.733	1.075 (0.600-1.925)	0.809
TSat90 categories				
<1.2%	1		1	
1.2-12%	1.158 (0.896-1.249)	0.506	1.285 (0.873-1.891)	0.204
>12%	1.296 (1.027-1.636)	0.029	0.811 (0.404-1.629)	0.556

Adjusted for sex, age, BMI, smoking status, hypertension, CHD, type 2 diabetes, BPH, and hyperuricemia.

AHI, apnea-hypopnea index; OSA, obstructive sleep apnea; TSat90, percentage of nighttime with oxygen saturation <90%.

### 3.4 Relationship Between AHI and Cancer Incidence Stratified by Age and Sex

In the subgroup analysis by age, after correcting for sex, BMI, smoking status, hypertension, coronary heart disease, and diabetes, Cox regression analysis demonstrated that the risk of cancer incidence was enhanced as the AHI increased among patients aged <65 years (adjusted HR: 1.019, 95% CI: 1.007–1.031, *P*=0.002). Further, cancer incidence was significantly higher in the severe OSA group (adjusted HR: 2.825, 95% CI: 1.358–5.878, P=0.019; **Table 5**). Meanwhile, subgroup analysis by sex presented no significant correlation between sex and AHI (*P*>0.05) when AHI was included in the multivariate Cox regression analysis either as a continuous variable or as a ranked variable (**Table 6**).

**Table 5:** Multivariate Cox regression analysis of the relationship between cancer incidence and OSA severity stratified by age.

OSA categories	Age ≥65 years		Age <65 years	
	Adjusted HR (95% CI)	*P*	Adjusted HR (95% CI)	*P*
AHI (continuous)	1.004 (0.996-1.013)	0.298	1.019 (1.007-1.031)	0.002
Non-OSA	1	–	1	–
Mild OSA	1.015 (0.656-1.571)	0.945	1.023 (0.490-2.136)	0.952
Moderate OSA	1.138 (0.717-1.806)	0.583	0.511 (0.177-1.480)	0.216
Severe OSA	1.271 (0.796-2.031)	0.316	2.825 (1.358-5.878)	0.019

AHI, apnea-hypopnea index; OSA, obstructive sleep apnea.

**Table 6:** Multivariate Cox regression analysis of the association between cancer incidence and OSA severity stratified by sex.

OSA categories	Male		Female	
	Adjusted HR (95% CI)	*P*	Adjusted HR (95% CI)	*P*
AHI (continuous)	1.007 (1.000-1.015)	0.059	1.015 (0.999-1.031)	0.074
Non-OSA	1	–	1	–
Mild OSA	0.934 (0.619-1.411)	0.747	1.040 (0.422-2.564)	0.932
Moderate OSA	0.999 (0.641-1.557)	0.998	0.899 (0.315-2.562)	0.841
Severe OSA	1.392 (0.905-2.141)	0.133	1.660 (0.620-4.447)	0.314

Adjusted for age, BMI, smoking status, hypertension, CHD, type 2 diabetes, BPH, and hyperuricemia.

AHI, apnea-hypopnea index; OSA, obstructive sleep apnea.

## 4 Discussion

Support for the correlation between OSA and the risk of cancer incidence remains scarce, particularly in China. In this study of patients with OSA followed up for a median of 9.1 years, the median time to cancer diagnosis after the start of sleep monitoring was 5.4 years. By the end of follow-up, the incidence of all types of cancer was 6.3%, encompassing 6.6% of patients suffering from OSA. The risk of cancer incidence increased as the AHI increased, particularly in patients undergoing severe OSA aged <65 years. After adjusting for confounders, neither AHI nor TSat90 were related to cancer mortality. Lung cancer ranked first in incidence and cancer mortality among new-onset cancers. Based on our knowledge, this is a primarily large retrospective study to evaluate cancer incidence and mortality in an OSA cohort in mainland China.

The cancer incidence in previous studies on OSA varies. The lowest was only 3.5% from an insurance database (15), whereas the highest was 31.5% in a community-based resident study with 20-year follow-up (22). Cancer incidence ranged from 5.1% to 6.5% in three retrospective studies from hospitals (13, 14, 16), while a prospective cohort study reported an incidence rate of 8.2% (26). A recent meta-analysis showed that OSA is associated with an increased overall cancer incidence (17). In the current study, the incidence rate within a median follow-up of 9.1 years was similar to the three studies mentioned above at 6.3%. The top three new-onset cancers were lung, colorectal, and prostate. The incidence rate was the highest for lung cancer at of 1.5%, and this is higher than that currently known for Canada (0.6%) (16), France (0.8%) (26), and Taiwan Province of China (0.3%) (27).

In one meta-analysis, the three most common cancers in OSA patients were prostate, breast, and lung (17). According to studies on OSA and lung cancer, sleep apnea and nocturnal hypoxemia can enhance the incidence of lung cancer (28). Meanwhile, an increased severity of OSA is also a risk factor for shorter overall survival in lung cancer patients (29). Lung cancer ranks first in incidence and mortality in China owing to the large smoking population and cooking methods that produce high amounts of fumes (30). This may be an important influencing factor in lung cancer having the highest incidence rate in the current study.

AHI and TSat90 are the main markers for OSA, and their predictive value for cancer incidence has been inconsistent among studies. In a Spanish study (13), a higher severity of OSA and a longer duration of TSat90 were related to a higher cancer incidence in male patients aged <65 years (13). A prospective cohort study by Justeau et al. in France (26) revealed that TSat90 was associated with all-cancer incidence. In contrast, in Israel, Brenner et al. (14) concluded an obviously higher cancer risk in OSA patients with an AHI >57. Meanwhile, a Canadian study (16) failed to discover the correlation between AHI and cancer incidence. In addition, a meta-analysis also revealed a higher risk of cancer in patients with moderate-to-severe OSA (18). In the current work, OSA severity was positively associated with the incidence of cancer.

The risk of cancer increased as the AHI increased, but it was not associated with either TSat90 or the oxygen desaturation index, in contrast with the reports above (13, 26). Intermittent hypoxia, the hallmark feature of OSA, can generate oxidative stress and reactive oxygen species during periodic reoxidation, which is a key distinction between intermittent hypoxia and chronic continuous hypoxia (31). TSat90 does not directly reflect this intermittent hypoxic state. AHI is also affected by a small decrease in oxygen saturation or even a complete absence of desaturation at the onset of respiratory events, neither of which can fully replace this intermittent hypoxic state.

Age and sex are other important factors that influence OSA and cancer incidence. In the general population, the older the age, the higher the risk of cancer (32). There are also differences in cancer incidence by age in the OSA population. Several studies (20, 22, 26) found an enhanced occurrence of cancer in OSA patients aged >60 years. Nevertheless, a study conducted by Brenner et al. (14) from Israel demonstrated that in comparison with the non-OSA group, OSA patients aged <45 years and with AHI >57 had a 3.7-fold higher risk of cancer. The present study also proved that patients suffering from severe OSA aged <65 years had an obviously higher cancer risk, similar to the findings of Brenner et al. (14).

However, the number of OSA patients included in each cohort, the differences in the number of OSA patients by age, and the presence of multiple confounding factors limit the findings of each study to varying degrees. Sex is another factor of concern. Studies by Brenner et al. (14) and Sillah et al. (20) showed that men with OSA had a significantly lower risk of suffering from cancer than did women. However, Sillah et al. (20) included rates for different sexes in their statistical methods for estimating males and females, and their findings were inconclusive. A meta-analysis showed that cancer incidence in female OSA patients was 4.0%, slightly higher than the 3.5% incidence in male OSA patients (17), and males accounted for 80.7% of cases in our cohort. However, the number of cases of sex-related cancer types, such as breast and gynecologic tumors, was significantly lower, and there existed no obvious difference in the occurrence of all types of cancers between men and women.

In addition, OSA is a significant risk factor for cancer-related mortality (33). Animal models have shown that intermittent hypoxia can increase melanoma cell growth, necrosis, and lung metastasis (12) and promote cancer cell invasiveness (34). Cancer mortality was higher among OSA patients than among non-OSA patients in a Wisconsin cohort (23). A Spanish cohort (19) found that TSat90 was associated with cancer mortality in patients aged <65 years. Community data from Busselton (22) showed that moderate-to-severe OSA was an independent risk factor for all-cause mortality and tumor mortality. A study by Huang et al. (27) also found that the severity of OSA was associated with an increased risk of death in patients diagnosed with stage III and IV lung cancer.

The present study results showed that neither AHI nor TSat90 were in significant association with cancer mortality, although OSA aggravated all-cause mortality in patients. Besides the confounding factors associated with OSA itself, additional factors have influenced studies on OSA and cancer mortality risk. The risks of all-cancer mortality, cancer type, malignancy, and treatment modality are often difficult to include in the analysis. The increase in cancer mortality may also be due to the enhanced occurrence of cancer (23). In addition, cancer mortality rates differ across countries with different levels of medical care and health insurance policies (35). This can affect the accuracy of the results.

The strengths of this study are that the cohort had complete long-term follow-up and comprehensive data from the Centers for Disease Control information system. In contrast, most cohorts analyzed OSA and cancer incidence or mortality separately. This study explored the occurrence and mortality of OSA and cancer in patients with new-onset cancers. In addition, the analysis of cancer mortality included all patients who developed new-onset cancers after sleep monitoring.

However, the present study also has limitations. First, the records of our telephone follow-up on whether OSA patients were treated with constant positive airway pressure therapy (CPAP) showed that 116 patients answered “yes”, accounting for 3.1%, and 3,670 patients answered “no”, accounting for 96.9%. The number of patients treated with CPAP is too small, and the information obtained is not sufficient for analysis. Thus, information about CPAP, which could influence the effect of OSA on the risk of cancer development (26), was not included in this study. Further prospective studies are needed to analyze the impact of OSA treatment on cancer in the future. Second, we did not have data on other potential confounding variables, such as alcohol consumption and occupation, which affect sleep apnea (36, 37) and cancer risk (38, 39). Finally, this was a retrospective single-center study, and the Guangdong Provincial People’s Hospital is a general hospital focusing on geriatric medicine and lung cancer research. The high proportion of elderly male patients in the cohort resulted in fewer female-specific cancer types, such as breast and gynecologic cancers. Considering that both OSA and cancer are major chronic diseases, further prospective large-scale studies are needed to elucidate their relationship.

In conclusion, the severity of OSA is related to the incidence of cancer in the Chinese population. The risk of cancer incidence increases as the AHI increases, especially in patients suffering from severe OSA aged <65 years. Mortality was higher in OSA patients than in non-OSA patients.

## Data Availability Statement

The raw data supporting the conclusions of this article will be made available by the authors, without undue reservation.

## Ethics Statement

The studies involving human participants were reviewed and approved by the Ethics Committee of Guangdong Provincial People’s Hospital. The ethics committee waived the requirement of written informed consent for participation.

## Author Contributions

HX and QO designed the study. HX, ML, YX, LW, GP, and BL performed sleep monitoring and follow-up. HX, QS, and JC analyzed the data. HX, LW, and SZ wrote the manuscript. All authors approved the final version of the manuscript.

## Funding

This work was supported by National Natural Science Foundation of China (Grant No. 81870077).

## Conflict of Interest

The authors declare that the research was conducted in the absence of any commercial or financial relationships that could be construed as a potential conflict of interest.

## Publisher’s Note

All claims expressed in this article are solely those of the authors and do not necessarily represent those of their affiliated organizations, or those of the publisher, the editors and the reviewers. Any product that may be evaluated in this article, or claim that may be made by its manufacturer, is not guaranteed or endorsed by the publisher.
